# Influence of film mulching on soil microbial community in a rainfed region of northeastern China

**DOI:** 10.1038/s41598-017-08575-w

**Published:** 2017-08-16

**Authors:** Wenyi Dong, Pengfei Si, Enke Liu, Changrong Yan, Zhe Zhang, Yanqing Zhang

**Affiliations:** 1 Institute of Environment and Sustainable Development in Agriculture, Chinese Academy of Agricultural Sciences, Beijing, 100081 PR China; 2 Key Laboratory for Prevention and Control of Residual Pollution in Agricultural Film, Ministry of Agriculture of the People’s Republic of China (MOA), Beijing, 100081 PR China; 3 College of Land and Environment, Shenyang Agriculture University, Shenyang, 110866 PR China; 4 Liaoning Academy of Agricultural Sciences, Shenyang, 110161 PR China

## Abstract

Information about the effect of plastic film mulching (PFM) on the soil microbial communities of rainfed regions remains scarce. In the present study, Illumina Hiseq sequencer was employed to compare the soil bacterial and fungal communities under three treatments: no mulching (NM), spring mulching (SM) and autumn mulching (AM) in two layers (0–10 and, 10–20 cm). Our results demonstrated that the plastic film mulching (PFM) application had positive effects on soil physicochemical properties as compared to no-mulching (NM): higher soil temperature (ST), greater soil moisture content (SMC) and better soil nutrients. Moreover, mulching application (especially AM) caused a significant increase of bacterial and fungal richness and diversity and played important roles in shaping microbial community composition. These effects were mainly explained by the ST and SMC induced by the PFM application. The positive effects of AM and SM on species abundances were very similar, while the AM harbored relatively more beneficial microbial taxa than the SM, e.g., taxa related to higher degrading capacity and nutrient cycling. According to the overall effects of AM application on ST, SMC, soil nutrients and microbial diversity, AM is recommended during maize cultivation in rain-fed region of northeast China.

## Introduction

Plastic film mulching (PFM) has been used for crop production worldwide since the 1950s, and its use has increased, particularly in arid and semi-arid areas^1^. Over 30 million acres of agricultural land worldwide were covered with plastic mulch as of 1999, and an estimated 1 million tons of mulch films are used annually in the agricultural sector^2^. Maize is the most important field crop in cold and arid regions of northeast China and occupies the largest cultivated area. Low temperature and water shortage are the major limiting factors for maize production^3^. The application of film mulching technology has expanded the maize planting area and enhanced maize production by 10–15 billion kilograms per year, which is the equivalent of 5–8% of the total national maize output in China^4^.

The soil microbial community plays a crucial role in nutrient cycling, maintenance of soil structure and its diversity is a sensitive indicator of soil quality that can reflect subtle changes and provide information for evaluation of soil function^5^. The determination of factors that influence microbial community composition under field conditions has significantly increased our understanding of how management affects crop quality, disease ecology, and biogeochemical cycling^6^. Thus, research on the effects of soil management on soil microbial communities has become a fundamental aspect of sustainable agriculture.

Studies have confirmed that PFM is generally associated with physical and chemical changes in soil characteristics, such as decreases in the amount of water loss caused by evaporation^7^, enhancement of soil temperatures^8^ and changes in soil nutrient availability^1^. Previous studies mainly focused on the impacts of PFM on soil moisture, soil structure, soil nutrition and crop yield, while their influences on the soil micro-ecological environment and the evolution of soil microbial community were neglected^9^. Just as different groups of microorganisms vary in their ability to adapt to the various soil environmental conditions, changes on soil microbiology under PFM application are expectable^10^. Li *et al*.^11^ demonstrated that PFM application increased the soil microbial activity in a spring wheat field, but the extent of this effect depended on how long the mulching was maintained during the growing season. In a study conducted in a maize cropping system, PFM showed higher microbial biomass carbon (C) and nitrogen (N) and several enzyme activities of C, N, and phosphorus (P) cycling compared with no mulching (NM)^12^. However, the detailed effects of continuous PFM on soil microbial communities and the link between these effects and the soil environment remain unclear. Particularly, Chen *et al*.^13^ indicated that bacterial communities in soils that underwent various mulching treatments obviously differed from those in soils that received NM in an orchard, and they were associated with different soil physicochemical conditions. Liu *et al*.^14^ found that PFM treatment could significantly change the community composition of arbuscular mycorrhizal fungi under a spring wheat cropping system in a temperate semi-arid region. Soil fungi and bacteria exhibit different community dynamics^15^; thus, to understand soil ecosystem processes, it is essential to address fungal and bacterial communities simultaneously^16^. However, how both bacterial and fungal communities respond to the microclimate conditions generated by PFM has not been elucidated together in a single study so far.

The western area of Liaoning Province is a typical semi-arid rain-fed agricultural region of China in which the limited rainfall cannot meet the agricultural production demands. Previous research has demonstrated that PFM treatments, particularly autumn mulching (AM) and spring mulching (SM), were the most optimal technical models for increasing water use efficiency and maize yield in these areas^17^. In the local agricultural production, SM application was conducted in spring of the present year until the harvest, while AM application was conducted in autumn of the previous year until the harvest. Therefore, the length of film mulching time under AM and SM were fixed and different. However, the effects of different mulching treatments on the microbial community structure have not been assessed in a comparative manner. Since PFM application generates artificial microclimatic conditions which may have an influence on the microbial diversity and abundance in soil, the main objective of the present work was to evaluate the impacts of different PFM approaches on the soil microbial community during rainfed maize production. With this aim, the phylum to genus levels of the composition and diversity of bacterial and fungal communities under the soil of two mulching treatments and NM were compared. Moreover, the relationships of the microbial community structure and diversity with the physicochemical properties of the PFM soils were analysed. We hypothesized that (1) both the AM and SM mulching treatments would lead to a higher soil microbial diversity and a restructuring of soil microbial communities compared with no-mulching treatment, and that (2) the variation in bacterial and fungal communities might also be associated with soil environmental conditions, e.g. soil organic carbon (SOC)and soil moisture content (SMC). To our knowledge, this is the first study to investigate bacterial and fungal communities under different PFM treatments.

## Materials and Methods

### Site description

Field experiments were conducted at the Fuxin Agricultural Environment and Farmland Conservation Experimental Station of China’s Ministry of Agriculture in Fuxin County (42°09′N, 121°46′E), Liaoning Province, and northeast China, which has a semi-arid climate. From 2011 to 2014, the mean annual air temperature was 7.2 °C, the average annual precipitation was 490 mm, and 73% of this precipitation fell during the maize growing season (from April to September). The average potential evaporation was 1830 mm. The soil at the experiment site is classified as Udalfs according to the USDA soil taxonomy system. Based on the investigation and analysis before our experiment, the soil properties at the top 20 cm were as follows: bulk density, 1.35 g · cm^−3^; pH, 6.95; soil organic carbon, 6.1 g · kg^−1^, total nitrogen (TN), 0.456 g · kg^−1^; total phosphorus (P), 0.66 g · kg^−1^; total potassium (K), 2.46 g · kg^−1^; available N, 196.6 mg · kg^−1^; available P, 136.1 mg · kg^−1^ and available K, 62.2 mg · kg^−1^.

### Field experiment design

In 2011, the experiment was composed of three different PFM treatments: (1) no mulching (NM); (2) spring mulching (SM): mulching in spring of the present year (from April 8th to the harvest); (3) autumn mulching (AM), mulching in autumn of the previous year (from November 10th to the harvest). Each treatment plot (10 m long × 5 m wide) was replicated three times and laid out in a randomized complete block design. In PFM treatments, the whole land surface (all ridges and furrows) was covered with polyethylene film (colorless and transparent, each strip was 0.008 mm thick and 1.2 m wide) for the soil, and the amount of film was approximately 75 kg · ha^−1^.

At the end of April, a maize cultivar “Zhengdan 958” was sown at a plant density of 60,000 plants ha^−1^ using a hole-sowing tool. All plots were harvested at the end of September. In 2014, the maize yields in different treatments were as follows: NM (12470 ± 363.059 kg · ha^−1^), SM (12024 ± 312.692 kg · ha^−1^) and AM (14280 ± 330.327 kg · ha^−1^).The harvest was followed by a fallow period until sowing time. Each plot was supplied with 240 kg N · ha^−1^ (urea, N, 46%), 140 kg P · ha^−1^ (calcium superphosphate, P_2_O_5_, 12%) and 190 kg K · ha^−1^ (potassium sulfate, K_2_SO_4_, 45%) at sowing.

### Sampling collection and analysis

After four years of continuous mulching treatments, soil samples were collected from the plots at the time of harvest on September 18th, 2014. In the PFM plots, the soil cores were sampled by penetrating the plastic film. The mulching materials on the sampling points were carefully removed during the collection of soil samples^18^. For all plots, soil at five randomly selected locations was sampled in two layers (0–10 cm and 10–20 cm, referred to as the surface and sub-surface, respectively) using an auger with a 5 cm internal diameter and then mixed as one sample. Every year after harvest, the plastic films were gathered and recycled. All of the fresh soil samples were air-dried and sieved twice, using 2.0 mm and 0.25 mm meshes. Each sample was then divided into two portions: one portion was stored at 4 °C for chemical analysis, and the other portion was stored at −80 °C for DNA extraction.

#### Soil chemical property analysis

The soil chemical properties were measured using the methods described by Bao^19^. Soil moisture content (SMC) and soil temperature (ST) were monitored in the fields using EcH_2_O (Decagon Devices, WA, USA) soil moisture and temperature sensors that were attached to a data logger from sowing to harvest, soil bulk density (BD) was determined using the core method. The SOC level was measured with a K_2_CrO_7_-H_2_SO_4_ oxidation method. The TN was measured using the Kjeldahl digestion method. The dissolved organic carbon (DOC) content of the filtered 0.5 M K_2_SO_4_ extracts from the fresh soil. Concentrations of DOC and total dissolved nitrogen (TDN) were determined with a TOC analyser (Liqui TOC Elementar, Vario Max, Germany). Concentration of dissolved organic nitrogen (DON) was calculated as the difference between the TDN reading and the combined ammonium-N and nitrate-N source reading.

#### DNA extraction and PCR amplification

Microbial DNA was extracted from 0.5 g soil samples using a Power Soil DNA Kit (MOBIO Inc., Carlsbad, CA) according to the manufacturer’s protocols. PCR was performed with the Takara ExTaq PCR kit (Takara Shuzo, Osaka, Japan). The V3-V4 regions of the bacterial 16S rRNA gene were amplified using the following PCR conditions: 95 °C for 3 min; followed by 30 cycles of 95 °C for 30 s, 55 °C for 30 s, and 72 °C for 45 s; and a final extension at 72 °C for 10 min. The following PCR primers were used for this amplification: 338 F 5′-ACTCCTACGGGAGGCAGCA-3′ and 806 R 5′-GGACTACHVGGGTWTCTAAT-3′. The region of the fungal ITS ribosomal gene was amplified using the following PCR conditions: 95 °C for 3 min, followed by 34 cycles at 95 °C for 30 s, 55 °C for 30 s, and 72 °C for 45 s and a final extension at 72 °C for 10 min. The primers ITS1F 5′-CTTGGTCATTTAGAGGAAGTAA-3′ and ITS2GCTGCGTTCTTCATCGATGC-3′ were used^20^.

### Illumina HiSeq sequencer

The amplicons were extracted from 2% agarose gels and purified using the AxyPrep DNA Gel Extraction Kit (Axygen Biosciences, Union City, CA, USA.) and quantified using QuantiFluor™-ST (Promega, USA.) according to the manufacturer’s instructions. Each sample was quantified using a Qubit3.0 Fluorometer (Life Technologies, Grand Island, NY), then pooled at equal concentrations and diluted to create one sample. Paired-end with a read length of 2 × 250 bp was carried out on an Illumina HiSeq platform according to standard protocols. Raw FASTQ files were de-multiplexed and, quality-filtered using QIIME 1.17 with the following criteria: (i) exact primer matching, two nucleotide mismatches in primer matching, and reads containing ambiguous characters were removed; and (ii)initial quality control measures removed any sequence with a consensus fold-coverage <5, average quality score <25 (50 bp rolling window). The reads that could not be assembled were discarded. All sequences with ambiguous base calls were discarded (bacteria: 933972 reads in total and 825614 effective reads, fungi: 1117315 reads in total and 1088110 effective reads). Effective sequences were normalized for the following analysis. The raw readings were deposited into the NCBI Sequence Read Archive (SRA) database with bioproject accession number PRJNA350374: SAMN05949267 to SAMN05949284 for bacteria and SAMN05949285 to SAMN05949302 for fungi.

### Statistical analysis

All of the results were reported as the means and standard error (SE). Our data passed three assumptions before analysis: 1) there were no significant outliers; 2) the dependent variable was normally distributed for each combination of the groups of the two independent variables; 3) our data variance was homogeneous for each combination of the groups of the two independent variables. The effects of mulching treatments, soil layers, and their combined interaction on fungal and bacterial assemblage diversity and richness and soil properties were determined by two-way analysis of variance (ANOVA) (SPSS Statistics, Version 18 IBM Corp., Armonk, NY, USA), and statistically significant differences were tested using Duncan’s multiple range test. Data analysis followed the steps below: 1) test interaction to see if there are combination effects. If so, see which combinations are different from the others and by how much, if no interactions, test the factors separately. If a factor is important (large F), decide which of its means are different and by how much. In every soil layer, one-way analysis of variance (ANOVA) and Duncan’s multiple comparisons were performed to identify differences in the microbial relative abundances among the three treatments. A difference at P < 0.05 was considered statistically significant.

Operational taxonomic units (OTUs) were clustered with a 97% similarity cut off using Uclust version v1.2.22q, and chimeric sequences were identified and removed using UCHIME. Completeness of the sampling effort was evaluated using Good’s rarefaction curves (Supplemental Fig. S1) and coverage (Supplemental Table S1). The phylogenetic affiliation of each 16S rRNA gene sequence was analysed with RDP Classifier (http://rdp.cme.msu.edu) against the SILVA (SSU123) 16S rRNA database using a confidence threshold of 60%. The α-diversity analysis, which was based on Mothur v.1.30.1, was conducted to reveal the Chao1, Shannon and Simpson diversity indices. In species richness estimation, the Chao1 index is the most commonly used, and which is based upon the number of rare classes (i.e., OTUs) found in a sample. In an ecosystem, the higher the Chao1 index presented, the richer the system. The Shannon index and the Simpson index were defined as being the most sensitive to changes in the importance of the species in the sample. Hence, to characterize species diversity in a community, we adopted two of the most widely used indices (Shannon index and Simpson’s index). The Shannon index measured the average degree of uncertainty in predicting the species of an individual chosen at random from a collection, and the value increases as the number of species increases and as the distribution of individuals among the species becomes even. The Simpson’s index indicates species dominance and reflects the probability of randomly choosing two individuals that belong to the same species. It varies from 0 to 1, and the index increases as the diversity decreases. For ß-diversity analysis, Principal Coordinates Analysis (PCoA) was performed to assess the similarities of the samples’ community memberships. The goal of PCoA is to decrease the dimensionality while preserving the pairwise dissimilarity values as much as possible in the data set^21^.

In our analysis, the weighted UniFrac distance metrics (based on phylogenetic structure) were used to generate PCoA using the principal_coordinates.py command in QIIME^22^. To determine the key factor(s) affecting microbial diversity, stepwise multiple regression analysis was applied using the probability criteria of P < 0.05 to accept and P > 0.1 to remove a variable from the analysis. Direct ordination was either by canonical correspondence analysis (CCA) or redundancy discriminate analysis (RDA), depending on the length of the detrended correspondence analysis (DCA) axis (where an axis of > 4.0 = CCA and an axis of < 4.0 = RDA, ‘decorana’ function in vegan). The initial DCA results demonstrated that the data exhibited unimodal rather than linear responses to the environmental variables. Therefore, CCA was carried out using the “cca” function in the vegan package of R (version 2.15.1) to assess the relationships between environmental parameters and microbial community structures^23^. The significances of environmental factors (included SMC, ST, BD, SOC, DOC, TN and DON) were assessed using the “envfit” function, which after determining r^2^ for environmental variables uses a permutation procedure to define the significance of each environmental variable (999 permutations) on all axes conjointly. Variables with no significant differences were removed from the analysis.

## Results

### Soil physical and chemical parameters

The SMC was always higher in the mulched plots than in the NM plot, and the differences were more obvious in middle growing season (Supplemental Fig. S2). For different treatments, the AM plots had higher SMC than that under the other two treatments for most sampling dates during the whole growing stage. The average SMC under the AM treatment in surface and subsurface soil was 4%, and 6% higher than that in the SM and NM treatments, respectively. Similarly, the ST in the soils was slightly higher in the PFM treatments than those in the NM treatments during most of the growing periods. The ST ranged from 17.6 to 28.9 °C, with an average of 22.7 °C under AM in surface soil, and from 17.4 to 26.5 °C, with an average of 21.7 °C in subsurface. Throughout the growing season, the ST in the AM was, on average, 1.5 °C and 2.0 °C higher than that in the SM and NM, respectively.

For the investigated soil properties after the harvesting stage the average TN, DOC and DON contents in AM were highest and were approximately 58, 39 and 101% higher than those of the NM treatment, respectively (Table 1). However, compared with the NM, the mulching treatments had no significant influence on SOC and BD contents (P > 0.05). The SMC, ST, BD, SOC and DOC were found to be significantly different between two layers (P < 0.05). Specifically, the SMC and BD at surface were significantly higher than that in subsurface. In contrast, the SOC and DOC surface were obviously less than that in subsurface. Moreover, there were no significant differences were observed in TN and DON between two layers.

**Table 1:** Effects of different mulching treatments on the physical and chemical properties of the soils sampled at two layers.

layer	Treatment	SMC (%)	ST (°C)	BD (g · cm^−3^)	SOC (g · kg^−1^)	TN (g · kg^−1^)	DOC (mg · kg^−1^)	DON (mg · kg^−1^)
Surface	NM	14 (0.6)	20.1 (0.1)	1.28 (0.04)	6.3 (0.0)	0.58 (0.01)	41.7 (4.9)	82.2 (6.5)
	SM	17 (0.2)	20.9 (0.1)	1.27 (0.04)	6.2 (0.1)	0.49 (0.07)	48.7 (3.9)	126.7 (29.8)
	AM	19 (0.2)	21.4 (0.1)	1.28 (0.05)	6.5 (0.0)	0.63 (0.11)	56.9 (2.1)	227.6 (31.3)
Subsurface	NM	25 (0.1)	19.5 (0.1)	1.48 (0.02)	4.9 (0.2)	0.41 (0.09)	34.4 (3.7)	102.4 (6.2)
	SM	26 (0.9)	19.8 (0.1)	1.44 (0.03)	5.1 (0.3)	0.30 (0.06)	43.9 (1.4)	124.3 (47.8)
	AM	28 (1.0)	20.3 (0.1)	1.38 (0.03)	5.3 (0.1)	0.63 (0.11)	48.6 (3.4)	141.7 (27.1)
Mulching effect(M)								
	NM	19 c	19.8 c	1.38 ns	5.6 ns	0.40 c	38.1 b	92.3 b
	SM	21 b	20.3 b	1.36 ns	5.6 ns	0.50 bc	46.4 b	125.5 ab
	AM	24 a	20.9 a	1.33 ns	5.9 ns	0.63 a	52.8 a	184.6 a
Layer effect(D)								
	surface	17 b	20.8 a	1.28 b	6.3 a	0.57 ns	49.1 a	145.5 ns
	subsurface	26 a	19.9 b	1.44 a	5.1 b	0.45 ns	42.3 b	122.8 ns
Significance								
	Mulching (M)	**	**	ns	ns	*	**	*
	Layer (L)	**	**	**	**	ns	*	ns
	M × L	**	ns	ns	ns	ns	ns	ns

Values are means (with standard error in parentheses). The values in mulching effect or layer effect were given the mean under each mulching treatment or soil layer. SMC: soil moisture content; ST: soil temperature; BD: bulk density; SOC: soil organic carbon; TN, total nitrogen; DOC, dissolved organic carbon; DON: dissolved organic nitrogen; Values within columns followed by the same letter do not differ at <0.05. ns: not significant. *Significant at 0.05 level. **Significant at 0.01 level.

### Richness and diversity of bacterial and fungal communities

Bacterial and fungal richness and diversity are shown in Table 2. The ANOVA results showed that both the mulching treatment and soil layer affected bacterial and fungal community richness (Chao1) and the highest Chao1 values for both bacterial and fungal communities were observed under AM treatment. For diversity indices, bacterial and fungal communities displayed different trends. Specifically, the Shannon index of bacterial communities in soils with mulching (AM: 7.31 and SM: 7.16, respectively) were significantly higher than those in soils with NM (6.75), and the Simpson indices (AM: 0.002 and SM: 0.003, respectively) were clearly lower than those of soils with NM (0.007). In addition, the soil layer effect on the bacterial diversity indices was not significant. In contrast, both the mulching treatments and the soil layer significantly affected fungal richness and diversity (P < 0.05), and AM treatment soil showed the greatest values of richness and the Shannon index, as well as the lowest Simpson index.

**Table 2:** Effects of different mulching treatments on bacterial and fungal α-diversity.

Layer	Treatment	Bacterial			Fungal		
		Chao1	Simpson	Shannon	Chao1	Simpson	Shannon
Surface	SM	7153 (58)	0.003 (0.000)	7.16 (0.00)	1393 (57)	0.0678 (0.008)	4.00 (0.02)
	AM	7433 (140)	0.002 (0.000)	7.35 (0.01)	1610 (101)	0.049 (0.002)	4.10 (0.04)
	NM	6126 (57)	0.007 (0.001)	6.87 (0.13)	1139 (31)	0.099 (0.005)	3.59 (0.03)
Subsurface	SM	6406 (51)	0.003 (0.000)	7.17 (0.03)	1240 (23)	0.082 (0.004)	3.78 (0.08)
	AM	6707 (82)	0.002 (0.001)	7.28 (0.02)	1343 (40)	0.069 (0.002)	3.93 (0.06)
	NM	5366 (131)	0.008 (0.000)	6.64 (0.22)	751 (91)	0.168 (0.010)	3.18 (0.02)
Mulching effect (M)							
SM		6780 b	0.003b	7.16 a	1317 b	0.059 c	3.38 c
AM		7070 a	0.002b	7.31 a	1477 a	0.074 b	4.01 a
NM		5746 c	0.007a	6.75 b	946 c	0.133 a	3.89 b
Layer effect (L)							
Surface		6904 b	0.004 ns	7.13 ns	1380 a	0.071 b	3.89 a
Subsurface		6159 a	0.004 ns	7.03 ns	1111 b	0.106 a	3.63 b
Significance							
Mulching (M)		**	**	**	**	**	**
Layer (L)		**	ns	ns	**	**	**
M × L		ns	ns	ns	ns	**	ns

Values are shown as the means (with standard error in parentheses). The values in mulching effect or layer effect were given the means under each mulching treatment or soil layer. *P < 0.05; **P < 0.01; ns, not significant.

### Treatment effects on bacterial and fungal ß-diversity

**Figure 1:**
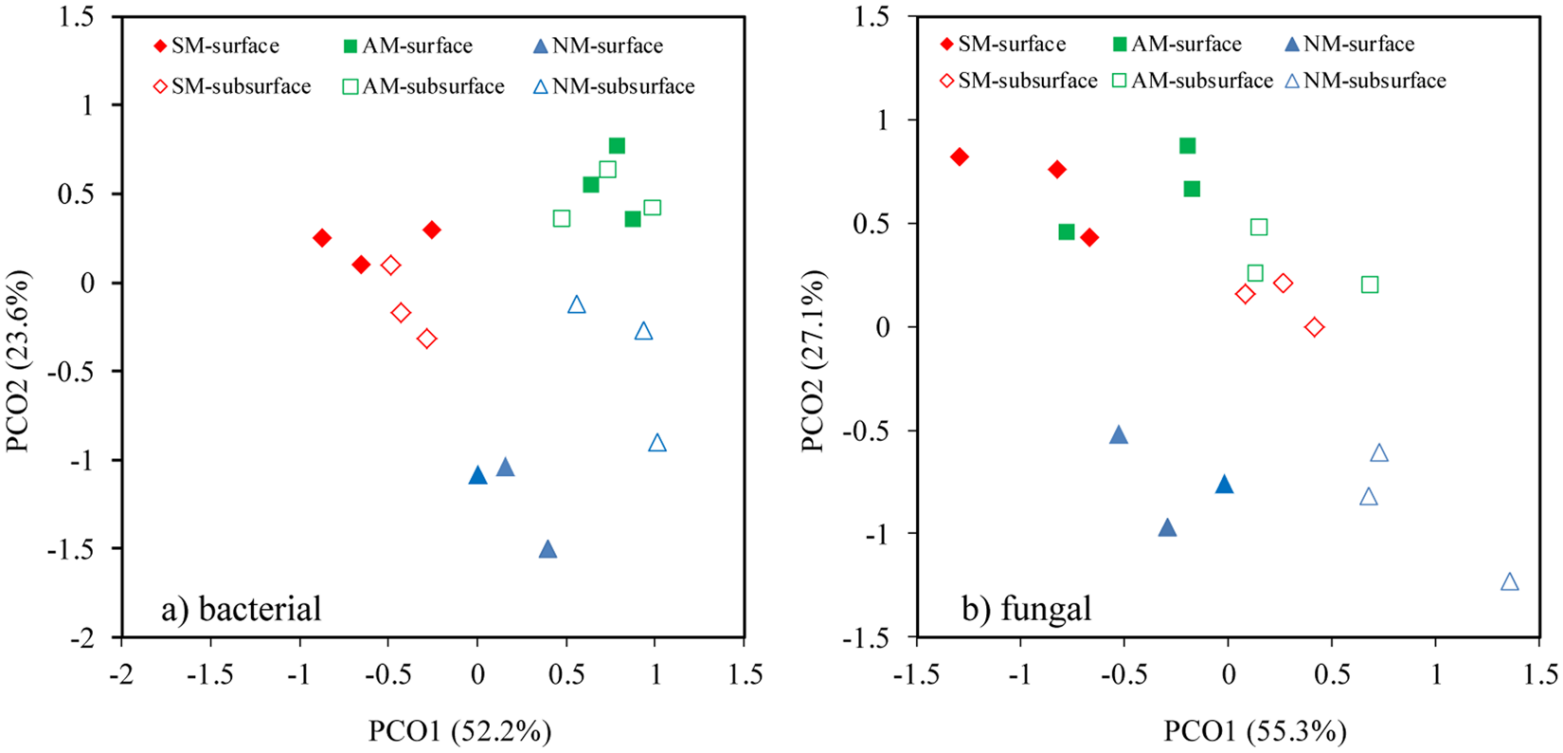
UniFrac-weighted PCoA plots of bacterial (**a**) and fungal (**b**) communities for different treatments in the two layers.

UniFrac-weighted PCoA based on the OTU composition also clearly demonstrated variations among the different soil samples, with the first two axes explaining 52.2% and 23.6% of the total variation for the bacterial and 55.3% and 27.1% for the fungi (Fig. 1), respectively. For bacteria, the NM treatment was distinctly separated from SM and AM treatments along the first component (PCoA1) in surface soil, while the AM treatment was separated from the SM and NM treatments along the second component (PCoA2) in the subsurface. For fungi, the NM treatment was clearly separated from the SM and AM treatments along the second component (PCoA2) in both layers. For the different soil layers, fungi exhibited more obvious separation than bacteria along the first component.

### Predominant bacterial and fungal taxa

#### Taxonomic composition of bacterial and fungal communities at the phylum level

**Figure 2:**
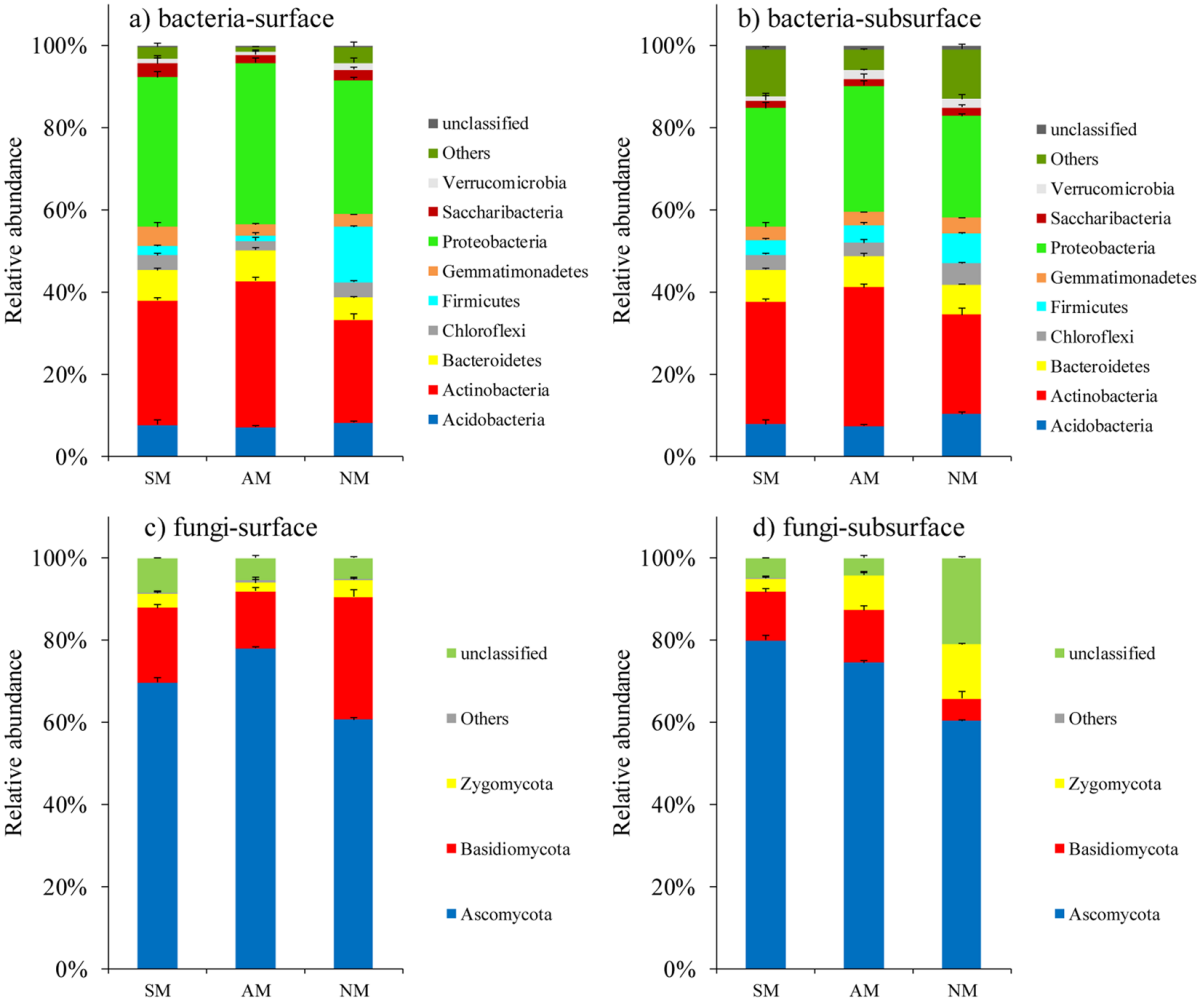
Taxonomic distributions of bacterial phyla in surface (**a**) and subsurface (**b**) soil samples and of fungal phyla in surface (**c**), and, subsurface (**d**) soil samples. Phyla having a mean relative abundance of at least 1% are shown individually, and less abundant phyla are grouped into other.

The phylum level composition of the bacterial and fungal communities varied among the different mulching treatments (Fig. 2). The bacterial representatives phyla *Proteobacteria* and *Actinobacteria* were dominant for all treatments in surface and subsurface soil communities. Comparing the differences in the abundance of these phyla, in surface soil, mulching treatment (especially the AM treatment) revealed a significantly higher abundance of sequences affiliated with *Actinobacteria*, *Bacteroidetes* and *Proteobacteria*, while samples from the NM treatment had significantly higher numbers of sequences related to *Chloroflexi* and *Firmicutes* (P < 0.05) (Supplemental Table S2). In subsurface soil, the mulching treatment samples had greater abundances of sequences affiliated with *Actinobacteria*, and *Proteobacteria* than the NM treatment samples. Similarly, the NM treatment samples had higher numbers of sequences related to *Chloroflexi* and *Firmicutes*. No pronounced differences in the relative abundances of the other four phyla (*Acidobacteria*, *Gemmatimonadetes*, *Saccharibacteria* and *Verrucomicrobia*) were observed between the communities for either soil layer.

Across all treatments in the two soil layers, the three most dominant fungal phyla were *Ascomycota*, *Basidiomycota* and *Zygomycota*, which accounted for more than 90% of the total fungal sequences (Fig. 2c,d). For fungi, *Ascomycota* were significantly more abundant in mulching samples (AM and SM) than in the NM samples for both the layers (P < 0.05). Nevertheless, the abundance of *Basidiomycota* in the NM samples was significantly higher than that in the AM samples in surface soil and lower in subsurface soil (P < 0.05).

#### Taxonomic composition of bacterial and fungal communities at the class level

**Figure 3:**
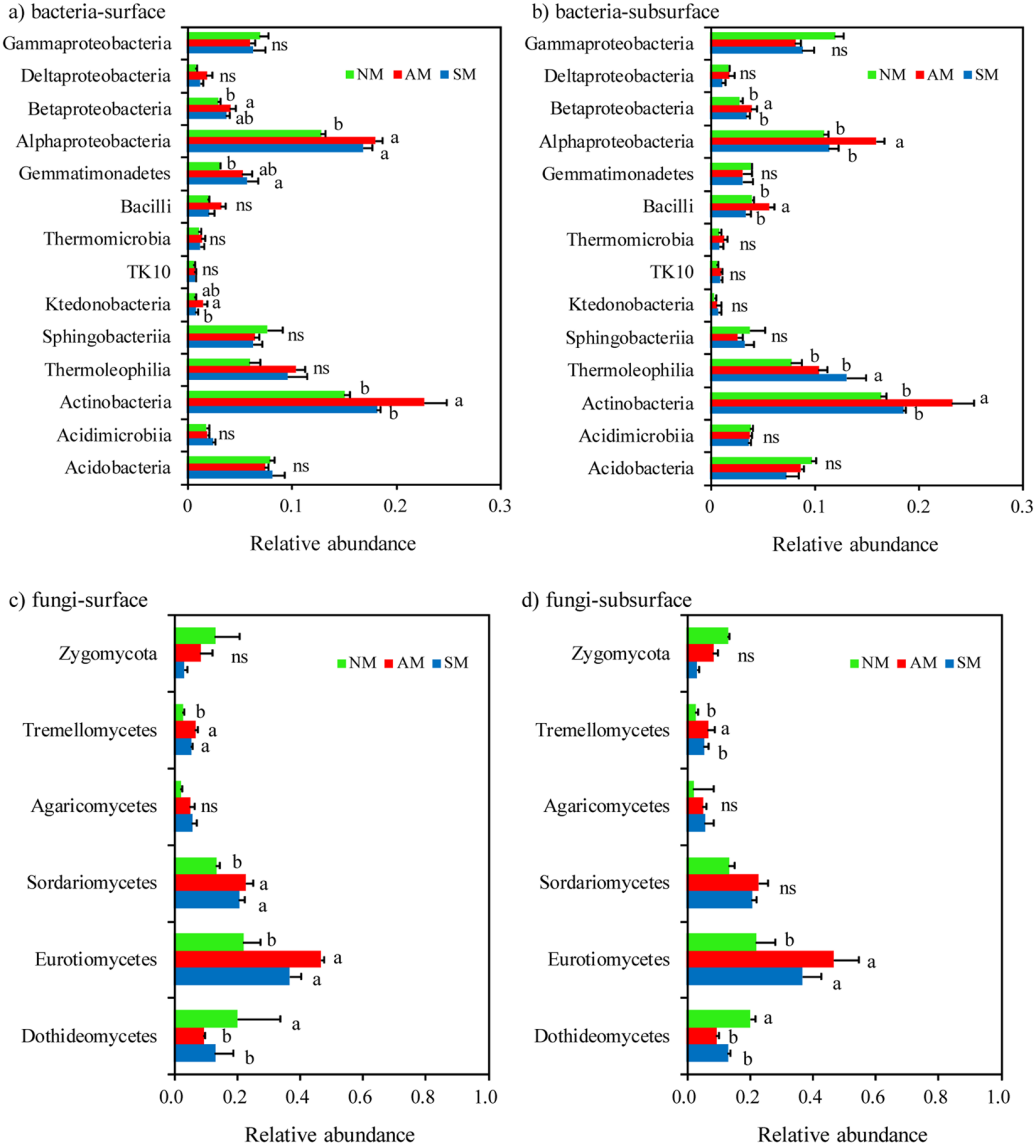
Taxonomic distributions of predominant classes of bacteria (**a**,**b**) and of fungal classes in surface (**c**), and, subsurface (**d**) soil samples. The different letter indicates significant difference at P < 0.05.

Patterns of taxonomic distribution became more apparent at the class levels. The five most abundant bacterial classes found in mulching treatment soil samples were *Actinobacteria*, *Alphaproteobacteria*, *Acidobacteria*, *Gammaproteobacteria*, and *Sphingobacteria* (Fig. 3). In the surface soil, the AM treatment samples had significantly greater abundances of sequences affiliated with *Actinobacteria* than other two treatments (P < 0.05). In the subsurface soil samples, the AM treatment samples had greater abundances of sequences affiliated with *Actinobacteria*, *Alphaproteobacteria*, and *Betaproteobacteria* than the other two treatment samples. Meanwhile, the SM treatment samples had higher numbers of sequences related to *Thermomicrobia* than other two treatments samples in the subsurface. For fungi, classes affiliated with *Eurotiomycetes*, *Dothideomycetes* and *Sordariomycetes* were the most abundant, consisting of greater than 70% of the classified fungal classes for both soil layers. The abundances of *Eurotiomycetes* were significantly higher in the mulching treatment samples than in the NM treatment samples (P < 0.05), whereas the abundances of *Dothideomycetes* were higher in the NM treatment samples than in the mulching treatment samples.

#### Taxonomic composition of bacterial and fungal communities at the genus level

**Figure 4:**
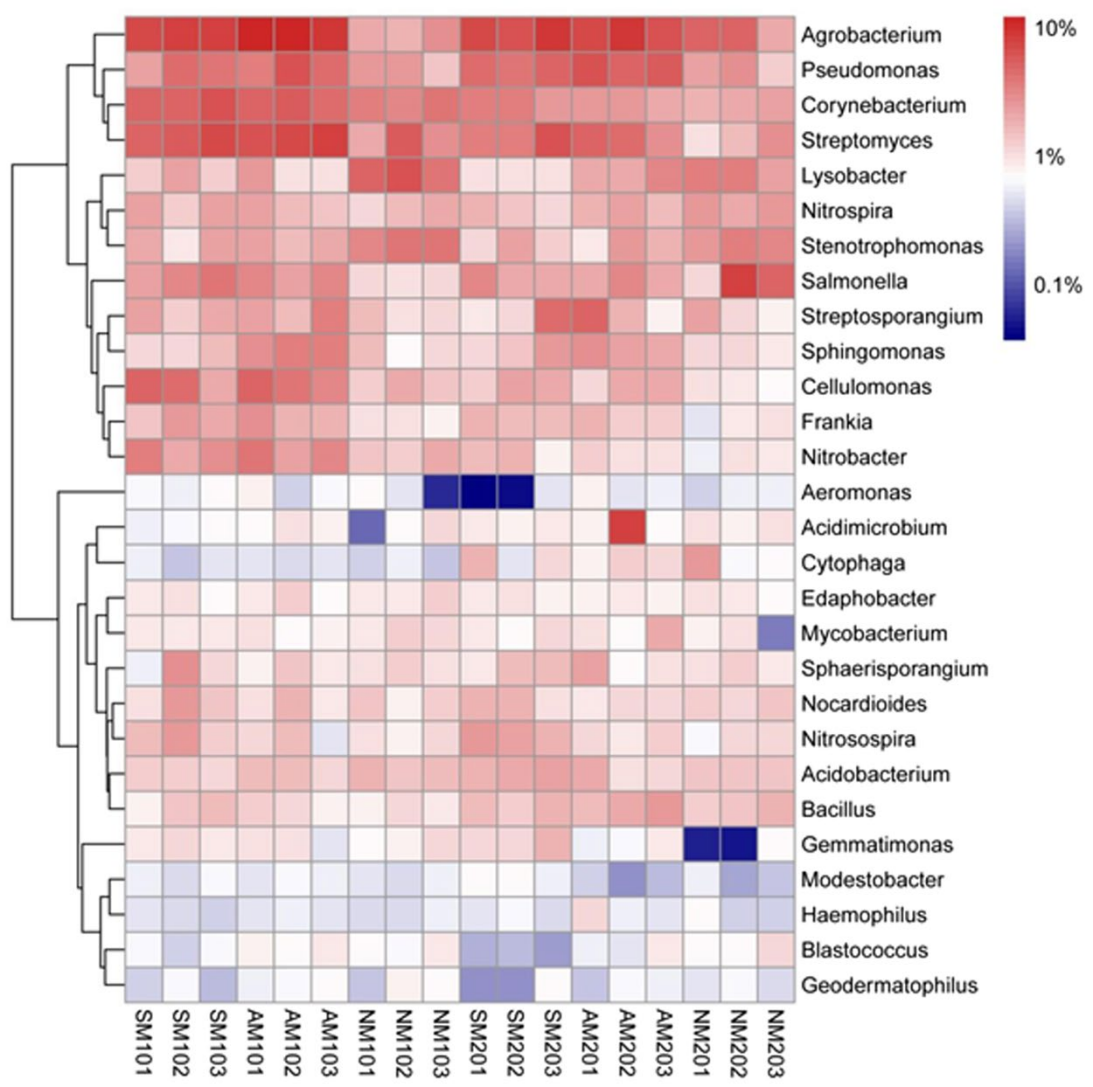
Heat map showing the abundance of bacterial genera in each sample (the genera with an average abundance greater than 0.5% in one group were defined as abundant). The colour intensity (log scale) in each panel shows the percentage of a genus in a sample; please refer to the colour key at the right bottom.

**Figure 5:**
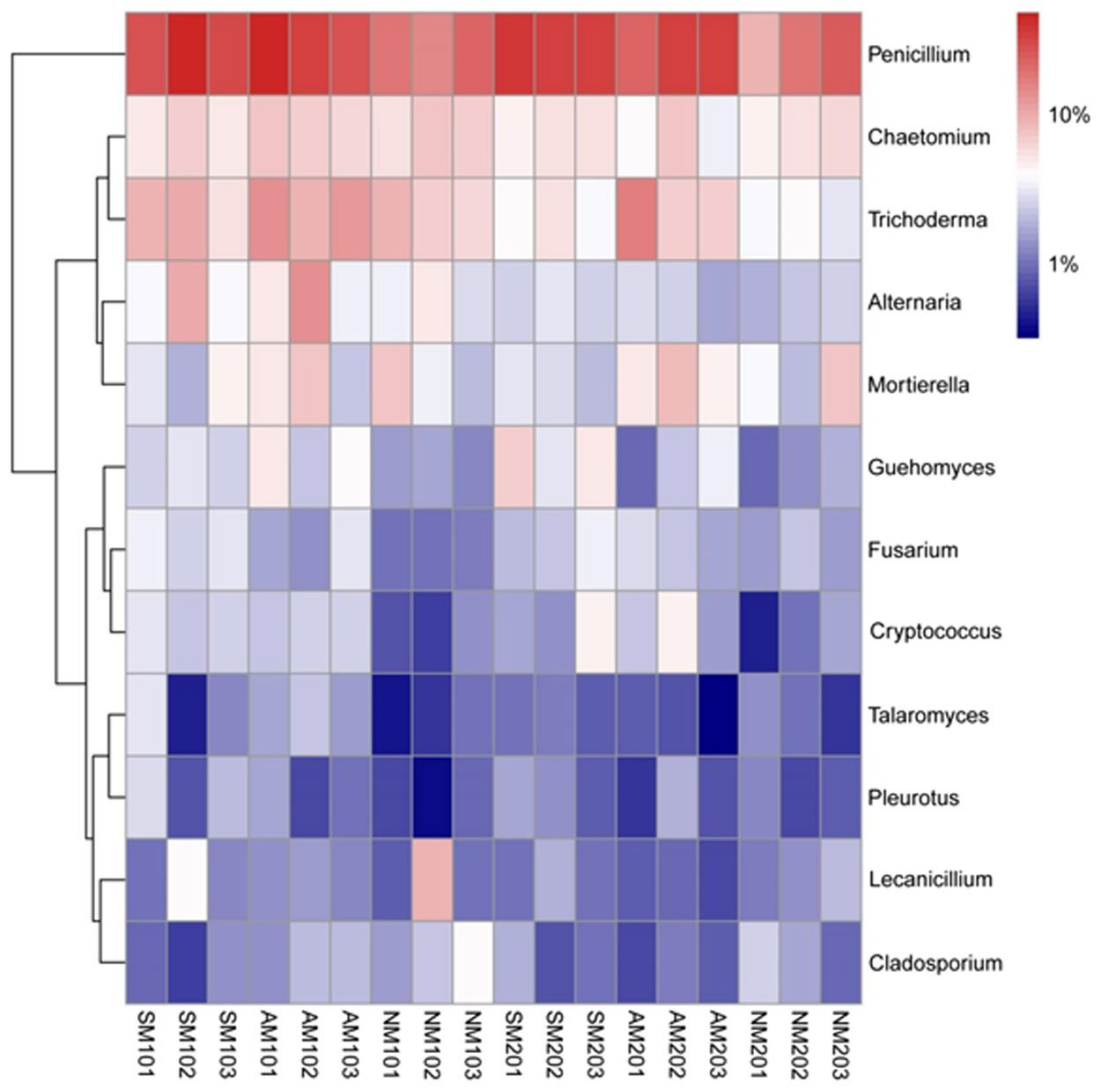
Heat map showing the abundance of fungal genera in each sample (the genera with an average abundance greater than 0.5% in one group were defined as abundant). The colour intensity (log scale) in each panel shows the percentage of a genus in a sample; please refer to colour key at the right bottom.

Based on relative abundance, five dominant bacterial genera were *Agrobacterium*, *Pseudomonas*, *Corynebacterium*, *Streptomyces* and *Lysobacter* (Fig. 4). The relative abundances of *Streptosporangium* and *Cytophaga* in surface and subsurface soil samples had different change trends: *Streptosporangium* was more enriched in the surface soil samples, whereas *Cytophaga* was more abundant in the subsurface soil samples. Microorganisms from the bacterial genera *Corynebacterium* (*Actinobacteria*), *Frankia* (*Actinobacteria*), *Nitrobacter* (*Proteobacteria*), *Cellulomonas* (*Actinobacteria*), *Streptomyces* (*Actinobacteria*), *Agrobacterium* (*Proteobacteria*) and *Pseudomonas* (*Proteobacteria*) showed higher abundances in the AM and SM treatments soil samples (particularly AM) than that in the NM treatment soil samples. However, the relative abundances of *Lysobacter* and *Stenotrophomonas* were significantly higher in the NM treatment soil samples. Distinctions in the composition of fungal communities were also observed at the genus level (Fig. 5). The most abundant genera found in both layers were *Penicillium* (*Ascomycota*), *Chaetomium* (*Ascomycota*) and *Trichoderma* (*Ascomycota*). Comparing the differences in the proportion of all abundant genera, AM treatment soil samples had greater abundances of sequences affiliated with *Penicillium*, *Talaromyces*, *Trichoderma* and *Cryptococcus*, while SM treatment soil samples had higher numbers of sequences related to *Fusarium* than NM treatment soil samples. In contrast, NM treatment obviously enriched the abundance of *Cladosporium*. Moreover, the major genera were more abundant in surface soil relative to subsurface soil samples, except *Mortierella*, which presented higher abundances in subsurface soil samples.

### Relationship between the soil microbial communityα-diversity, composition and environmental parameters

A stepwise regression analysis was conducted to determine the correlation of soil environmental parameters with microbial communityα-diversity (Table 3). The results revealed that ST significantly affected bacterial and fungal α-diversity index, including bacterial Chao1, Simpson index and Shannon index, fungal Chao1, Simpson index and Shannon index. The SOC was also an important factor which clearly influenced bacterial Simpson index and fungal Chao1.

**Table 3:** The variables which were found by stepwise regression analysis to be correlated with bacterial and fungal α-diversity index.

	Dependents	Variables related	R^2^	Significance
Bacterial	Chao1	ST	0.771	<0.001
	Simpson	ST SOC	0.760	<0.001
	Shannon	ST	0.501	<0.001
Fungal	Chao1	ST SOC	0.794	<0.001
	Simpson	ST	0.677	<0.001
	Shannon	ST	0.666	<0.001

**Figure 6:**
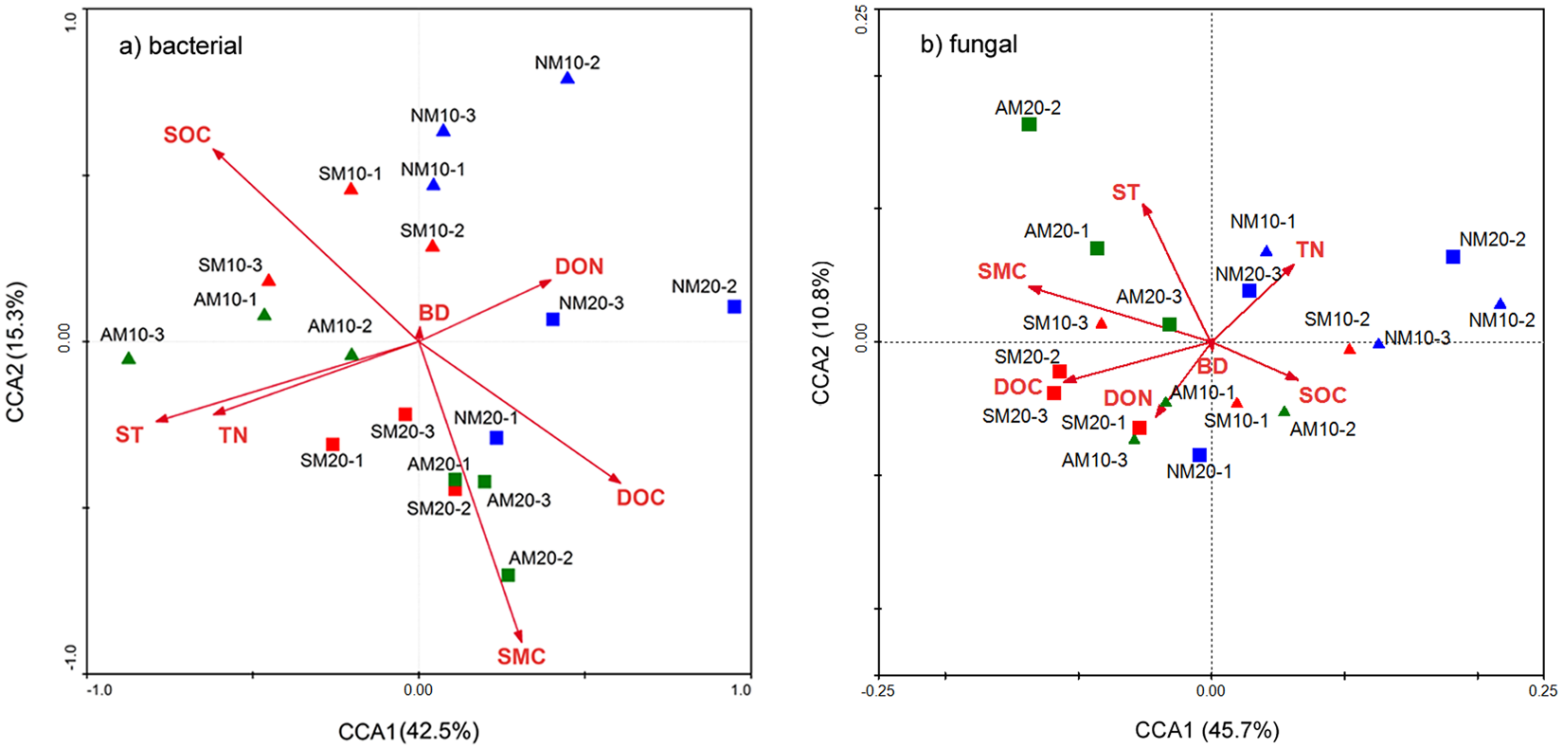
Canonical corresponding analysis (CCA) of bacterial (**a**) and fungal (**b**) community compositions with environmental variables. (10, surface soil 20, subsurface soil).

To further explore the correlation of soil environmental parameters with community structures of bacteria and fungi, we performed a canonical correlation analysis (CCA) based on the sequencing data (Fig. 6). Mantel test analyses indicated that SMC, SOC and ST were significantly correlated with soil bacterial communities. SMC (F = 5.016, P = 0.003) was the most significantly correlated variable with soil bacterial communities at the genus level, followed by the SOC (F = 4.789, P = 0.006) and ST (F = 4.633, P = 0.008). In addition, we also investigated the soil factors that separately influenced the bacterial community composition in different soil layers. In the surface soil samples, we found that the ST (F = 4.421, P = 0.009), SMC (F = 3.841, P = 0.021) and SOC (F = 3.403, P = 0.038) strongly influenced the bacteria community composition (Supplemental Fig. S3). In the subsurface soil samples, the SMC (F = 4.016, P = 0.016), DOC (F = 3.755, P = 0.028) and TN (F = 3.435, P = 0.036) were the main drivers of the bacterial community composition.

For fungi, SMC (F = 5.003, P = 0.004) had the strongest correlation with the community composition, followed by ST (F = 3.907, P = 0.012) and DOC (F = 3.455, P = 0.035). In surface soil samples, significant correlations were observed between the fungal community composition and SMC (F = 4.604, P = 0.008), ST (F = 3.823, P = 0.024) and SOC (F = 3.533, P = 0.034). In the subsurface, SMC (F = 4.886, P = 0.007), TN (F = 4.212, P = 0.010) and DOC (F = 3.854, P = 0.020) were significantly correlated with soil fungal community composition (Supplemental Fig. S4).

## Discussion

### Treatment effects on bacterial and fungal α-diversity

In our research, bacterial and fungal richness and diversity were higher (exhibited as higher Chao1 and, Shannon and lower Simpson indices) in the AM and SM treatment soil samples than in the NM treatment soil samples (Table 2). This finding suggested that the mulching treatments (especially the AM treatment) favored more complicated and diverse bacterial and fungal communities better than the NM treatment. Similarly, Wu *et al*.^24^ reported that a continuous PFM regime in paddy fields stimulated the development of diverse microbial communities better than the NM regime; these diverse microbial communities were attributed to higher amounts of high quality substrates. Moreover, it is important to recognize that the mulching treatment may increase functional diversity due to the observed increase in alpha diversity, which leads to a micro-ecological system that is more resilient to environmental change.

### Treatment effects on bacterial and fungal community composition

In our research, the PCoA revealed significant differences between the three mulching treatments and clearly grouped all of the samples separately, confirming that the dominant soil microbes differed significantly in their relative abundances among different treatments. The soil in the AM and SM treatments (especially the AM treatment) had significantly higher abundances of the dominant phyla *Proteobacteria* and *Actinobacteria* than the soils from the NM treatment (Fig. 2). Within the former phylum, the OTUs assigned to the bacterial classes *Alphaproteobacteria* were relatively frequent in both the AM and SM treatment soil samples. Similarly, Chen *et al*.^13^ found that the *Alphaproteobacteria* were greatly enriched in a grass mulching treatment compared with their richness in NM. As members of these classes have been shown to positively correlate with the utilization of a wide range of C compounds^25^, the increased abundances of these phyla in our study may be of particular importance in biogeochemical cycling of carbon and, as such, might be suitable to address the large variety of carbon sources under mulching conditions. Additionally, we found a higher abundance of *Actinobacteria* in AM treatment soil samples than in SM and NM treatment samples (especially in surface soil). Recent studies suggest an important role for *Actinobacteria* in soil metabolic functioning due to their involvement in the decomposition of organic materials^26^. Another striking pattern was the significant decreases in the relative abundances of the dominant phyla, *Chloroflexi* and *Firmicutes* in the AM and SM treatment soil samples. Members of *Chloroflexi* have also been found to tolerate extreme soil environments^27^. The higher frequency of *Firmicutes* in the NM treatment soil samples than in the mulching treatment soil samples may be related to their ability to produce endospores that are resistant to desiccation under NM environmental conditions^28^. Moreover, the *Firmicutes* exhibit rapid growth followed by abundant spore formation and is generally considered oligotrophic^29^.

Similar to the bacterial community, the fungal communities also responded differently to the mulching treatments, as the communities in the soil samples from the three treatments were clearly separated from each other. Moreover, *Ascomycota* and *Basidiomycota* were the most abundant fungal phyla in all of the soil samples, which are also in agreement with a previous study showing that *Ascomycota* and *Basidiomycota* accounting for more than 60% of the total sequences in soil samples as demonstrated by Illumina HiSeq sequencer^30^. Two dominant classes within *Ascomycota* (*Eurotiomycetes* and *Sordariomycetes*) increased in relative abundance under AM conditions (Fig. 3) and many more *Ascomycota* representatives contributed to the phylum-level composition shift in the surface soil samples. Regarding the abundances of the phyla in the soil samples, the enrichment of *Ascomycota* in soil samples may be associated with a higher disease suppression ability^31^. The relative abundance of *Basidiomycota* among treatments presented different trends in two soil layers, with a significant increase in NM treatment surface soil samples but a decrease in subsurface soil samples relative to the abundance in the AM treatment soil samples. This finding may suggest a higher sensitivity of this phylum to environmental changes^32^.

For bacteria, obvious trend in our study was that *Agrobacterium* and *Pseudomonas* were significantly enhanced in the mulching treatment, and were more evident in AM treatment. Similar result was reported by Bonanomi *et al*.^33^, and they attributed the predominance of these bacteria in soil under mulching to the anoxic environment. Moreover, the AM treatment resulted in the significant enrichment of *Frankia* and *Nitrobacter* genera. Members of *Frankia* are known as their ability to form N_2_-fixing root nodule symbioses with actinorhizal plants and *Nitrobacter* are generally considered playing an important role in the nitrogen cycle by oxidizing nitrite into nitrate in soil^34^. These findings illustrated that AM treatment has a significant ability to enhance microbes that drive soil nitrogen turnover in nutrient cycling under this agro-ecosystem. Many previous studies have demonstrated that *Corynebacterium* play important roles in degrading aromatic compounds and that *Cellulomonas* are generally associated with degrading cellulose using enzymes such as endoglucanase and exoglucanase^35^. Additionally, *Streptomyces* can produce a number of antibiotics and other bioactive natural products that suppress soil-borne diseases^36^. Thus, the higher frequency of OTUs assigned to these three genera indicated that the AM treatment markedly increased some beneficial bacterial species compared with the other treatments. However, in contrast, OTUs assigned to the genera *Lysobacter* and *Stenotrophomonas* were significantly less abundant in the AM and SM treatment soil samples than in the NM treatment soil samples. Consistent with this finding, Hayward *et al*.^37^ also found that exposure to natural light and rainfall conditions increased the number of these two genera due to their ability to more efficiently use lower levels of substrates than other bacterial genera.

Among fungi, the most dominant species were assigned to the genus *Penicillium*, which includes species that have been shown to play critical roles in the production of secondary metabolites or in the decomposition of organic matter^38^. Obvious differences in particular fungal taxa were also apparent across the soil samples from the three treatments. Acosta-Martínez *et al*.^32^ found that *Penicillium* species were ubiquitous soil fungi preferring moderate climates, which may explain their increased abundance in AM and SM treatment soil samples compared to NM. *Trichoderma*, *Talaromyces* and *Fusarium* were more frequent in AM and SM compared to that in NM treatment soil samples, and a previous study reported that members of these fungal groups were associated with degrading complex substrates, such as cellulose, chitin, and lignin-related compounds^31^.

### Links between the selected soil properties with the bacterial and fungal community composition

The correlation analysis indicates that specific changes in bacterial and fungal communities could be partially explained by soil characteristics. In the present research, the SMC in the mulching treatment was always higher than that of NM plot in the growing season and after harvest. These results are compatible to those of previous studies which were probably due to lower run-off and evaporation under mulching^39^. The daily mean ST in the mulched plots was higher than that in the NM plots during growing season in both layers. Similar observations have also been reported by Wang *et al*.^8^, and they considered that the effects of the PFM on ST was related to the plastic film absorbing and reflecting solar energy. For different treatments, the AM plots had higher SMC and ST than that under the other two treatments for most sampling dates during the whole growing stage. This may be due to AM was conducted in autumn of the previous year until the harvest. Therefore, AM application could achieve seasonal adjustment of soil moisture in rainfed area by preserving rainfall, improving soil water storage before sowing, and reducing invalid water loss during fallow period. It was reported that continuous use of plastic mulch may accelerate the decomposition of the SOC^40^. In this study, however, the SOC did not differ significantly between different mulching treatments at the sampling date in two layers. For other properties, our results suggest that the adoption of mulching significantly increased the content of TN, DOC and DON in each layer. Similar results have been observed in other studies, e.g. in the semiarid Loess Plateau of China, and they considered that the higher moisture content arose from PFM promoting soil nutrients accumulation^41^.

The stepwise regression analysis shows that ST was the most predominant factor in explaining the bacterial and fungal diversity under different mulching application (Table 3). Notably, ST has also been showed to have an important effect on soil bacterial and fungal community composition, as verified by the CCA model and Mantel test. This result is in general agreement with many other studies reported that temperature was the most influential factor on the soil microbial community, which were mainly due to ST could influence heterotrophic respiration by changing the activity of extracellular enzymes, the microbial respiration rate, and the substrate availability for the soil microbes^42, 43^. SMC was another major factor that influences the bacterial and fungal composition under different treatments, which was supported by our observation that SMC was significantly correlated with taxonomical composition (Fig. 6). A limited decrease in soil moisture may be a stressful process for some microorganisms, due to physical constraints that affect bacterial or fungal habitats^44^. As soil drying occurs, available water in pores becomes disconnected, slowing down diffusion of solutes and limiting substrate availability resulting in a decline in nutrient flow to microbes^45^. Apart from the ST and SMC, soil properties, such as the SOC, TN and DOC also significantly contributed to the variation in the bacterial and fungal community composition, which was probably explained by decomposing the crop residues with different chemical structure. Moreover, we found more genes and/or higher bacterial and fungal diversity in the AM than in the NM and SM, and one explanation is that mulching might cause a shift in the predominant microbial life history strategies, which favors more active, copiotrophic microbial groups^46^.

## Conclusion

In addition to the effects of mulching on soil properties, our research fills a gap in the understanding of the effects of different mulching treatments on abundance and composition of bacterial and fungal communities in soil under maize. Our results confirmed that mulching treatments, especially AM, played an important role in improving the SMC and ST conditions throughout the growing periods, and simultaneously increased the soil nutrients (e.g. TN, DOC and DON). The α-diversity indices (Chao1, Shannon and Simpson indices) confirmed the predicted increase in bacterial and fungal diversity as a result of both AM and SM treatments (especially AM). The positive effects of AM and SM on species abundances were very similar, while the AM harbored relatively more beneficial microbial taxa than the SM, e.g., taxa related to higher degrading capacity and nutrient cycling. Canonical correspondence analysis (CCA) revealed that the soil organic carbon (SOC) content was the most predominant main factor in explaining affecting the composition of the bacterial community, while soil moisture content (SMC) was the most important factor that determined in affecting the fungal community composition. Taken together, our data indicated that AM treatment rather than SM and NM treatments is a good practice for maintaining microbial diversity and altering microbial composition. The challenge in the future will be to better understand the variation in microbial patterns over time under different mulching treatments and elucidate the expression of functional genes within each community.

## Electronic supplementary material

Supplementary Information
