# Supplementary material for: Influence of film mulching on soil microbial community in a rainfed region of northeastern China
Source: Sci Rep. 2017 Aug 16;7:8468. doi: 10.1038/s41598-017-08575-w (PMC5559608; doi:10.1038/s41598-017-08575-w)
Supplement: Supplementary file 1 — Supplementary Information [file 41598_2017_8575_MOESM1_ESM.pdf]

Do soil bacterial and fungal communities in the rainfed region of northeastern China change under different film mulching systems?

Wenyi Dong[1,2+], Pengfei Si[3,4+], Enke Liu[1,2]*, Changrong Yan[1,2], Zhe Zhang[4] and Yanqing Zhang[1,2]

[1.] Institute of Environment and Sustainable Development in Agriculture, Chinese Academy of Agricultural Sciences, Beijing, 100081, PR China

[2.] Key Laboratory of Dryland Farming Agriculture, Ministry of Agriculture of the People's Republic of China (MOA), Beijing, 100081, PR China

[3] College of Land and Environment, Shenyang Agriculture University, Shenyang, 110866, PR China

[4] Liaoning Academy of Agricultural Sciences, Shenyang, 110161, PR China

*Corresponding author: happyterry2013@tom.com

+These authors contributed equally to this work

Supplemental Table S1 Coverage value for different soil samples across all soil treatments.

Sample ID	Coverage value	
	Bacterial	Fungal
SM0-10-1	0.93069	0.98861
SM0-10-2	0.95837	0.97520
SM0-10-3	0.96812	0.99652
AM0-10-1	0.97069	0.99357
AM0-10-2	0.96346	0.99232
AM0-10-3	0.96438	0.99716
NM0-10-1	0.91228	0.99236
NM0-10-2	0.93822	0.99443
NM0-10-3	0.93795	0.99412
SM10-20-1	0.96188	0.99738
SM10-20-2	0.95084	0.99639
SM10-20-3	0.85402	0.98079
AM10-20-1	0.88996	0.98723
AM10-20-2	0.93912	0.99182
AM10-20-3	0.93167	0.99190
NM10-20-1	0.95376	0.99707
NM10-20-2	0.97034	0.99631
NM10-20-3	0.93705	0.99266

Supplemental Table S2 Average relative abundances of bacterial phyla in surface (a) and,subsurface (b) soil samples and of fungal phyla in surface(c) and, subsurface (d) soil samples across all soil treatments.

Layer		Surface			Subsurface		
Treatments		SM	AM	NM	SM	AM	NM
Bacteria	Acidobacteria	7.70 (0.012) ns	7.06 (0.003) ns	8.23 (0.004) ns	7.72 (0.010) ns	7.41 (0.007) ns	10.31 (0.009) ns
	Actinobacteria	30.22 (0.007) b	35.64 (0.009) a	24.96 (0.016) c	29.92(0.006) ab	33.69 (0.024) a	24.22 (0.016) b
	Bacteroidetes	7.43 (0.004) b	7.49 (0.008) a	5.49 (0.002) c	7.85 (0.011) ns	7.54 (0.013) ns	7.33 (0.004) ns
	Chloroflexi	2.63 (0.002) b	2.27 (0.005) b	3.75 (0.004) a	3.60 (0.006) b	3.33 (0.003) b	5.18 (0.002) a
	Firmicutes	2.15 (0.005) b	1.40 (0.002) b	13.42 (0.004) a	3.63 (0.003) b	4.36 (0.003) b	7.12 (0.002) a
	Gemmatimonadetes	4.73 (0.012) ns	2.62 (0.001) ns	3.08 (0.001) ns	3.13 (0.003) ns	3.13 (0.003) ns	3.93 (0.006) ns
	Proteobacteria	36.53 (0.012) ab	39.35 (0.012) a	32.70 (0.006) b	29.04 (0.004) a	30.84 (0.003) a	24.75 (0.002) b
	Saccharibacteria	3.34 (0.004) ns	1.98 (0.003) ns	2.53 (0.003) ns	1.69 (0.014) ns	1.68 (0.007) ns	2.15 (0.006) ns
	Verrucomicrobia	1.24 (0.002) ns	0.76 (0.001) ns	1.65 (0.001) ns	1.11 (0.004) ns	2.09 (0.003) ns	2.03 (0.003) ns
	Others	2.80 (0.007)	1.12 (0.002)	3.94 (0.012)	11.40 (0.002)	5.03 (0.004)	12.10 (0.006)
	unclassified	0.23 (0.001)	0.30 (0.001)	0.24 (0.001)	0.89 (0.001)	0.90 (0.001)	0.87 (0.002)
Fungi	Ascomycota	69.64 (0.033) a	78.03 (0.046) a	60.63 (0.031) b	79.91 (0.022) a	74.60 (0.041)a	60.29 (0.134) b
	Basidiomycota	18.31 (0.066) ab	13.88 (0.036) b	29.95 (0.012) a	11.87 (0.013) a	12.78 (0.009) a	5.53 (0.003) b
	Zygomycota	3.46 (0.010) ns	2.12 (0.003) ns	4.19 (0.021) ns	3.20 (0.008) ns	8.38 (0.036) ns	13.13 (0.075) ns
	Others	0.20 (0.002)	0.58 (0.002)	0.27 (0.001)	0.20 (0.001)	0.11 (0.001)	0.10 (0.001)
	unclassified	8.39 (0.042)	5.38 (0.012)	4.96 (0.008)	4.82 (0.006)	4.13 (0.001)	20.94 (0.006)

The different letter in the same line indicates significant difference at $P<0.05$ among three treatments.

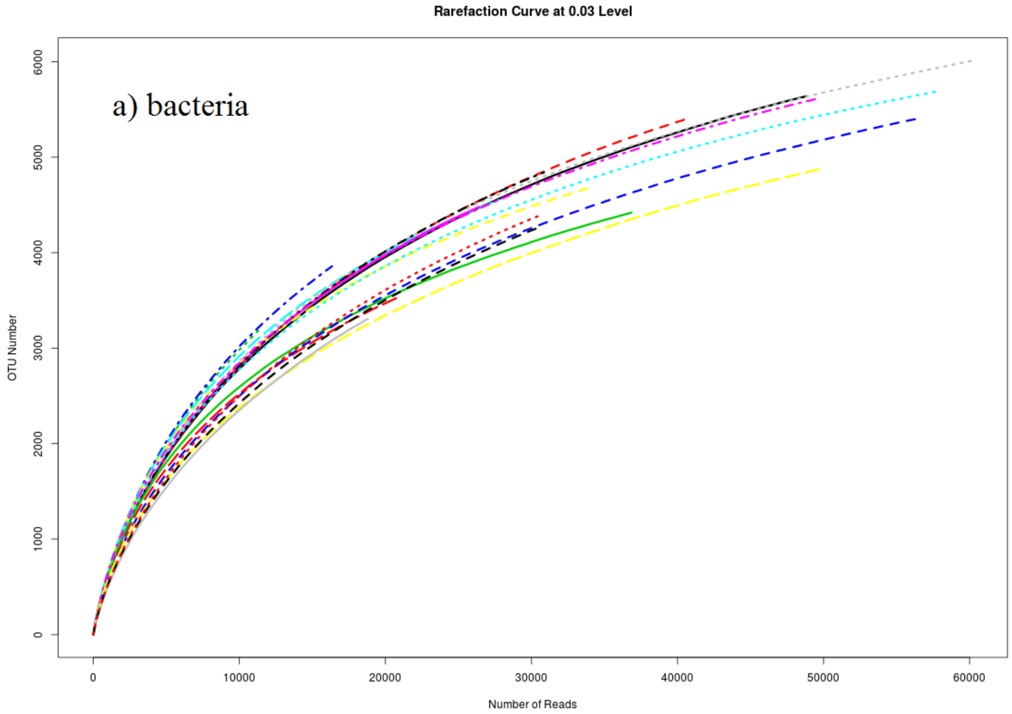

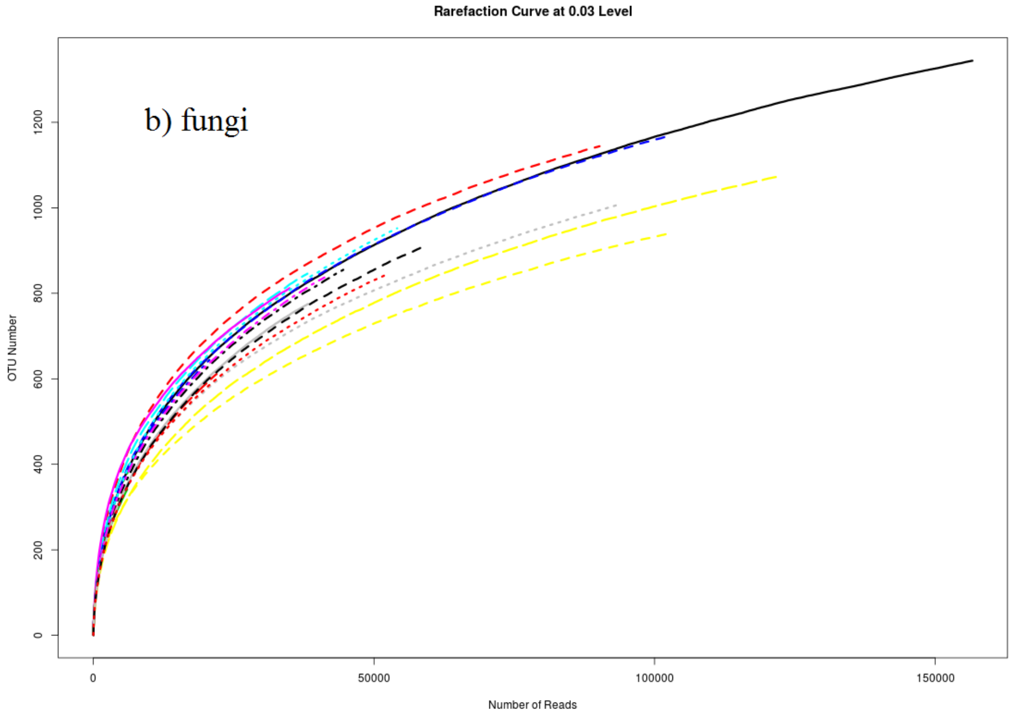

Supplemental Fig. S1 Rarefaction curves representing the numbers of operational taxonomic units (OTUs) versus the number of tags sampled from sequencing data at distances of 0.03

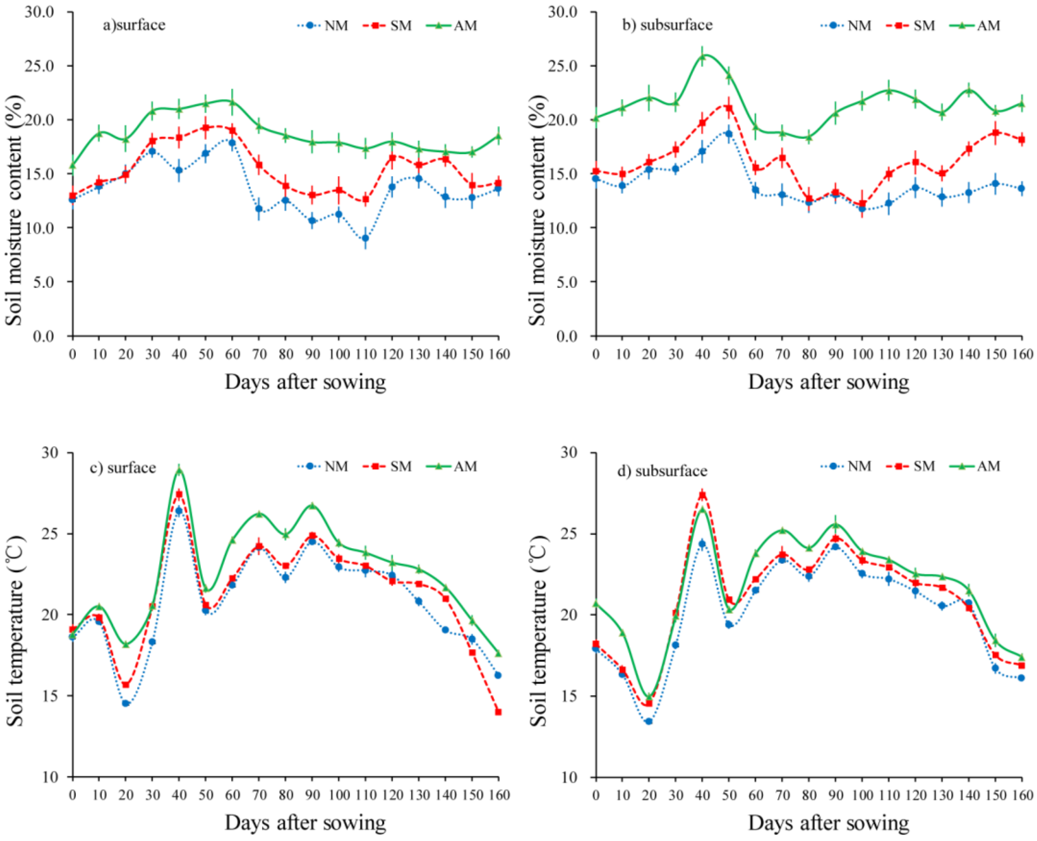

Supplemental Fig. S2 Changes in soil temperatures in surface (a) and subsurface (b) and moisture contents in surface (c) and subsurface (d) under different mulching treatments during maize cultivation.

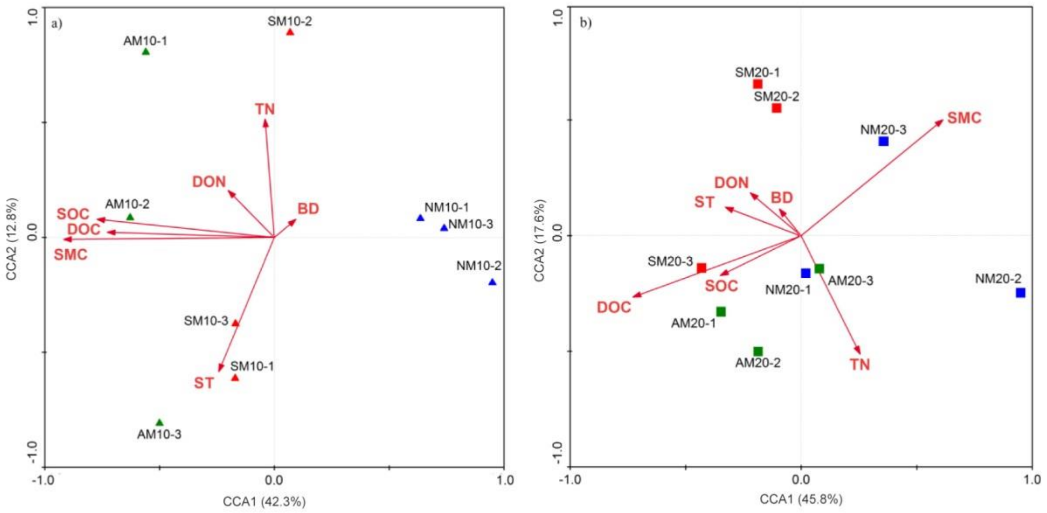

Supplemental Fig. S3 Canonical corresponding analysis (CCA) of bacterial community composition and environmental variables in (a) surface and (b) subsurface soil samples.

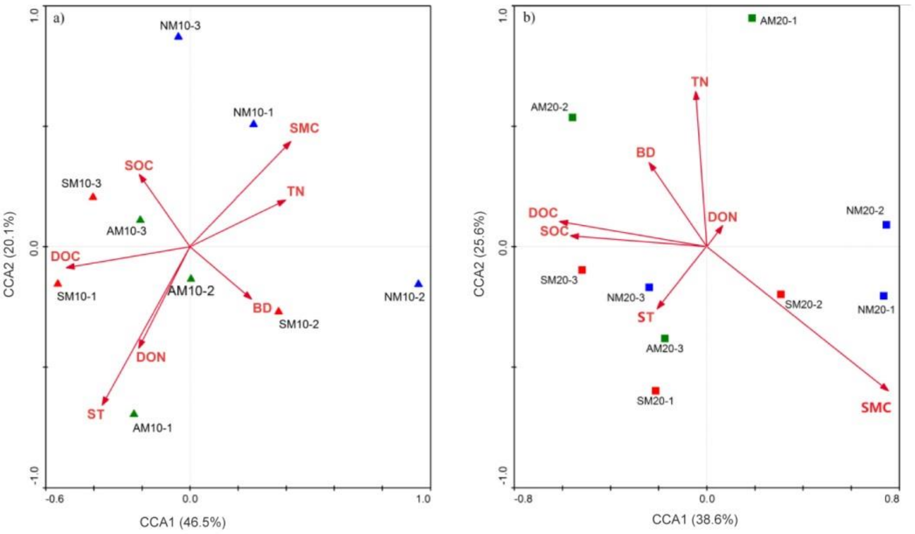

Supplemental Fig .S4 Canonical corresponding analysis (CCA) of fungal community composition and environmental variables in (a) surface and (b) subsurface soil samples.
